# The Fusion of Microfluidics and Optics for On-Chip Detection and Characterization of Microalgae

**DOI:** 10.3390/mi12101137

**Published:** 2021-09-22

**Authors:** Xinqi Zheng, Xiudong Duan, Xin Tu, Shulan Jiang, Chaolong Song

**Affiliations:** School of Mechanical Engineering and Electronic Information, China University of Geosciences, Wuhan 430074, China; zhengxinqi@cug.edu.cn (X.Z.); 904416366@cug.edu.cn (X.D.); tuxin@cug.edu.cn (X.T.); jiangshulan@cug.edu.cn (S.J.)

**Keywords:** optofluidics, microalgae, fluorescence, Raman spectroscopy, imaging-based flow cytometry, lipid, pigment

## Abstract

It has been demonstrated that microalgae play an important role in the food, agriculture and medicine industries. Additionally, the identification and counting of the microalgae are also a critical step in evaluating water quality, and some lipid-rich microalgae species even have the potential to be an alternative to fossil fuels. However, current technologies for the detection and analysis of microalgae are costly, labor-intensive, time-consuming and throughput limited. In the past few years, microfluidic chips integrating optical components have emerged as powerful tools that can be used for the analysis of microalgae with high specificity, sensitivity and throughput. In this paper, we review recent optofluidic lab-on-chip systems and techniques used for microalgal detection and characterization. We introduce three optofluidic technologies that are based on fluorescence, Raman spectroscopy and imaging-based flow cytometry, each of which can achieve the determination of cell viability, lipid content, metabolic heterogeneity and counting. We analyze and summarize the merits and drawbacks of these micro-systems and conclude the direction of the future development of the optofluidic platforms applied in microalgal research.

## 1. Introduction

There are about 200,000 to 800,000 microalgal species, spread widely in oceans, lakes, and rivers around the world [1]. As microalgae have unique properties of fast growth, high photosynthetic efficiency, short doubling time of multiplication and high yield per unit of volume [2], they play an important role in both the ecosystem and economics. Microalgae are rich in nutrients such as protein, Omega-3 polyunsaturated fatty acids, polysaccharides, and various vitamins [3,4], and microalgae are also key sources of aquatic animal food. The endogenous pigments within the microalgae have numerous commercial applications in the biotechnology, food, and pharmaceutical industries [5,6], such as labels in immunology experiments [7], natural food colorants [8,9], and nutraceuticals [10]. Moreover, it has recently been demonstrated that some microalgae species containing rich lipid could be an alternative to potentially replace fossil fuels as an emerging renewable biofuel, provided the microalgae species with high content of specific pigments and lipid can be with effectively screened out for selective cultivation [11,12]. On the other hand, eutrophication-induced microalgal multiplication could cause serious impacts on the water quality and the entire ecosystem due to the increasing industrial and agricultural production activities of humankind [13,14,15]. Considering the influences of microalgae in both economics and aquatic ecosystems, it is essential to monitor and analyze microalgae species dynamics in terms of the estimation of growth and activity, as well as the lipid and pigment quantification of microalgae.

Generally, detection or evaluation methods for the acquisition of microalgae information can be divided into two main categories: optical and non-optical. As non-optical methods, manual observation [2], chromatography (HPLC or GC) [16], targeting of specific molecules [17], and dielectric spectroscopy [18,19,20] have been widely applied to microalgal growth estimation, species identification, and lipid and pigment quantification. Conventionally, people can simply observe the chromaticity and turbidity of the culturing medium of microalgae to evaluate the growth rate of the different microalgae species. Destructive techniques usually involve chemical solvent to extract the pigments and lipid inside the cell for subsequent chromatographic analysis [16], which can be used to realize both identification and quantification of lipid and pigments. However, these methods rely greatly on experience-based assessment, and cannot be applied to single-cell analysis. Additionally, the procedure of chemical extraction could introduce damages to the cellular structure of the microalgae. Recently, lectin-cell surface polysaccharide binding [21,22] and antibody or oligonucleotide probes [23] have been employed with emerging biotechnologies for the detection of microalgae species with high sensitivity and specificity, but these methods require complicated operation procedures under strict experimental conditions, which could be time-consuming, labor-intensive and not cost-effective. Dielectric spectroscopy methods have recently emerged for the intracellular analysis of microalgae [24,25,26], and these methods can be used to evaluate the lipid level of the microalgae according to the permittivity and conductivity of microalgae cellular components [27,28]. Compared with other non-optical detection methods, dielectric spectroscopy has the advantages of less sample consumption, and rapid label-free and nondestructive measurement [29]. However, there is still the challenge that multiple factors such as ions and proteins within the cell can influence the effective dielectric properties of the cell, which may introduce crosstalk into the interpretation of detection results.

Optics-based detection methods have been flavored as powerful instruments for microalgal analysis and quantification due to their high sensitivity and specificity. Optical microscopy is one of the most popular methods used to identify microalgae according to the morphology of different microalgae species at micro-scale [30]. Different from the optical microscopy methods, the fluorescence-based methods and spectroscopy-based methods are mainly used to acquire intracellular information. The fluorescence-based methods can acquire multiple types of information according to the fluorescence intensity emitted by pigments via autofluorescence or stained dye fluorescence [31,32]. For example, the chlorophyll autofluorescence intensity can be used to assess microalgae cell activity and growth rate, and the fluorescence intensity of the BODIPY- or Nile red-stained lipid can reflect intracellular lipid amount. Spectroscopy-based techniques such as VIS/NIR spectroscopy [33,34,35], Raman spectroscopy [36] and Fourier transform infrared spectroscopy [37,38,39] have also been widely used for both identification and quantification of the pigments and lipid from the extracted spectral data. On top of this, the spectroscopy-based techniques can be applied for long-distance and real-time detection. Additionally, due to the rich pigments existing in microalgae cells, which absorb light at specific wavelengths, spectrophotometer-based methods can also be used for microalgae pigments and species quantitative analysis [40,41]. Compared with the non-optical methods, optics-based methods can simultaneously provide the information of both intracellular and morphological parameters.

However, the abovementioned optics-based methods still involve non-standardized manual operation, which would lead to a low throughput of detection and analysis. To enhance the throughput, commercial flow-cytometer can be used for microalgal detection [42], which usually makes use of the unique fluorescent and optical scattering properties of individual algae cells to achieve identification and counting of species. However, the traditional cytometer equipment is costly and bulky, which makes it unsuitable for application of on-site detection. Generally, traditional optical methods for microalgal detection are not suitable for continuous measurement and not applicable for an integrated platform of the multi-functional automated detection. Therefore, development of new methodologies and novel devices, which can offer cost-effective, high-throughput and non-destructive analysis of microalgae species has attracted increasing interests from research communities.

The past decades have witnessed the flourishment of microfluidic technologies, which have been developed for applications in fields of biotechnology, environment monitoring, analytical chemistry and pharmaceutics [43,44,45,46]. Microfluidic devices are widely favored due to their portable size for on-site analysis, their cost-effectiveness for disposable usage, and their high level of integration for multiple functions [47]. Recently, some researchers have been using microfluidic technologies for microalgae-relevant research, such as microalgal cultivation [48,49], strain selection [50] and harvesting [51], lipid extraction [52,53], and in situ measurement [54]. The microfluidic systems for microalgal detection mainly contain integrated optical and electrical architectures. The electrical integrated microfluidic systems mainly make use of electrical parameters such as capacitive [24], impedance [55,56] and dielectric properties [57,58] to characterize microalgae. Song et al. [55,56] developed resistive pulse sensors to monitor cell number, estimate cell size, and distinguish live cells from lysed cells in the PDMS microchannel, and the same group also identified activity of cells by a capacitive sensing device [24] according to the shift of capacitive response between live and dead cell. Fellahi et al. [59] quantified the lipid content of microalgae using dielectric spectroscopy based on a slight decrease of dielectric permittivity due to lipid content accumulation within the microalgae cell. The microfluidic detection platforms based on electrical characterizations usually involve complex procedures of fabrication of microelectrodes [60,61,62]. To ensure the accuracy of measurement, it is necessary to carefully arrange the positions of the microelectrodes and treat the sample in advance to control the medium composition.

In recent decades, optofluidic technologies, as a marriage between micro-optics and microfluidics, have emerged as powerful tools for biomedical and biochemical assays [63,64,65,66,67,68]. Recently, intensive research attention has been directed towards microalgal analysis using optofluidic technologies due to the unique advantages of high level of system integration, compactness and portability of the system, non-invasive manner of measurement, and capacity for multi-parameter extraction. Integrated photobioreactor has been proposed and demonstrated for on-chip d culture [69,70], with controllable light conditions for optimized microalgal photosynthesis to promote microalgal growth and lipid accumulation. Additionally, many researchers have reported integrated fluorescence sensing [71,72,73], spectroscopy [74,75,76], and other imaging techniques [28,36,77] on microfluidic platforms, which can be applied in microalgal detection to achieve acquisitions of both morphological and intracellular information with high throughput and low cost. However, few literature reviews have been reported on the recent progress of microalgae research using optofluidic technologies. In this review, we categorize and summarize the recent optofluidic systems and techniques for microalgal detection and characterization. Considering recent emerged microfluidic systems integrated with optical detection methods used for microalgal analysis with excellent performances, we specifically aim to summarize the effective optofluidic detection systems for microalgal analysis. In detail, we first introduce the optofluidic detection systems based on their categorization, such as fluorescence sensing, Raman spectroscopy and imaging flow cytometer, followed by comparisons and discussions of their merits and drawbacks, and then we conclude the possible directions of further development of microfluidic systems for microalgal detection and characterization. Table 1 summarizes the applications of the microfluidics for microalgal detection and quantification based on fluorescence, Raman spectroscopy and imaging-based flow cytometry.

## 2. Fluorescence-Based Sensing

As one of the most popular optical detection methods, fluorescence detection methods have been widely used in the detection and characterization of biological and biochemical samples in microfluidic chips [28]. Fluorescence of microalgae can be a result of either endogenous pigments, such as chlorophyll, phycoerythrin and phycocyanin inside the microalgae cell, or artificial staining dyes. The pigments in the cell can emit fluorescence with different wavelengths upon the excitation of the light, and different microalgae species have their unique fluorescence spectrums due to the different ratio of the pigments within the microalgae cells [91]. In addition, the endogenous fluorescence intensity can also be used to study cell viability [92]. Staining dyes, such as Nile red and BODIPY, can be used to label the algae cell for detection and quantification with application for lipid content estimation in microalgae species [93,94].

**Figure 1:**
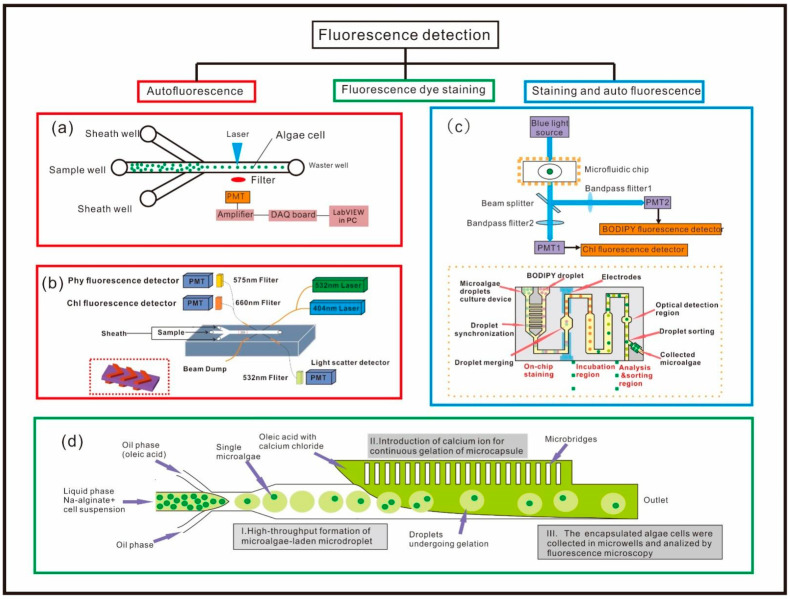
Optofluidic devices for microalgae detection based on fluorescence. (**a**) Schematic of the optofluidic system for single-cell activity study based on chlorophyll autofluorescence [71]. (**b**) Schematic of the optofluidic cytometer. The enlarged part is the chevron grooves on the PDMS substrate [73]. (**c**) Schematic of the optical setup and droplet-based microfluidic chip for cell sorting [82]. (**d**) Schematic of the microfluidic device for the generation of alginate hydrogel microcapsules containing microalgae cells [80].

It has been reported that chlorophyll fluorescence intensity is proportional to chlorophyll concentration within cells, which can be used to evaluate photosynthesis capacity and viability of the microalgae cell [95,96,97]. Wang et al. proposed a micro-device for investigation of individual microalgae cell activity based on the chlorophyll fluorescence [71], as shown in Figure 1a. This microfluidic chip consists of three microchannels, the laminar flows from two branch channels can force the microalgae cells in the main channel to align into a single line. A 488 nm laser diode was used as the excitation light to illuminate the sample cells. A photodiode was selected to measure chlorophyll fluorescence with output voltage corresponding to the fluorescence intensity. It can be used to identify dead cells and living cells by calibrating the fluorescence intensity. This device was able to distinguish six different species, among which the smallest cell detected by this biosensor was 3 μm. Hashemi et al. constructed a special microfluidic chip which can focus flows in two dimensions [73]. There are two grooves on the top and bottom of the channel, creating two symmetric sheath streams to wrap around a central core stream (Figure 1b). A 404 nm and a 532 nm laser were used to excite chlorophyll and phycobilins fluorescence, and three PMTs (photomultiplier tube) were used to record the fluorescence signals and light scattering signal. The differences in fluorescence signals can be used to reveal the different ratios of chlorophyll and phycobilins in microalgae species, and the differences in light scattering signals can be used to evaluate the size and shape of the microalgae cells. Four species of phytoplankton microalgae cells were identified and counted by this microflow cytometer, among which the smallest microalgae of the four is *Synechococcus* sp. with a size about 1 μm in diameter. Best et al. proposed a droplet-based microfluidic device for studying the growth dynamics of the microalgae cells, which has the capability to sort the cell encapsulated in the droplet according to the chlorophyll autofluorescence [79]. Microalgae cells cultured in sufficient and deficient nitrogen medium can possess different levels of chlorophyll content. Generally, microalgae cells grown in the medium with sufficient nitrogen have relatively higher levels of chlorophyll. Cells with high and low levels of chlorophyll were prepared in a ratio of 1:1 and injected into the microfluidic chip, and the microalgae cells were encapsulated into the droplets (one droplet contains one cell) by setting proper flow rates. As the droplet passed through the laser spot, the fluorescence signal from the microalgae cell was excited and received by the PMT and then recorded for analysis. Droplets containing higher levels of chlorophyll cells can lead to a higher signal peak above the pre-set threshold that can be treated as a ‘sorting event’. Upon triggering the ‘sorting event’, a sorting voltage was implemented to deflect the droplet into a ‘positive channel’, and those droplets containing lower levels of chlorophyll content were deflected into the other ‘negative channel’.

In addition to the autofluorescence measurements, some biocompatible staining dyes have also been used to evaluate the level of lipid content inside the cells. Holcomb et al. developed a device to study the influence of the nitrogen-depleted condition on microalgae cell lipid accumulation [81]. The device was able to realize both on-chip staining and culturing, and the experimental results showed that the microalgae cells grown in the nitrogen-limited conditions have a higher lipid content. Do-Hyun et al. proposed a microfluidic chip generating gelation droplets for the analysis of the lipid content of the microalgae cells with the aim of improving the throughput (Figure 1d) [80]. The stained cells suspended in the Na-alginate solution were encapsulated into droplets. The integrated micro-bridge structure was used to introduce calcium for continuous gelation of the microcapsule containing microalgae cells with sufficient spacing. After that, the trapped cells were collected into the microwells to acquire the fluorescent images by the fluorescence microscope. Normalized fluorescent intensities suggested that the intracellular lipid content is distinguishable in different microalgae species and the characterization of the lipid content on single cell resolution in the same species was also achieved. This method can significantly avoid evaporation of lipid and overlap of the fluorescence signal.

Autofluorescence combined with staining dye fluorescence has also been used as a powerful technique to simultaneously acquire information related to the endogenous pigments and lipid of microalgae cells, which can serve to estimate cell growth, photosynthetic efficiency and lipid accumulation at the same time. Hyun et al. proposed a microfluidic platform that can simultaneously achieve the analysis of the microalgal growth and lipid content (Figure 1c) [82]. This platform consisted of three interconnected modules: an on-chip staining region, an incubation region, and an analysis/sorting region. Microalgae cells suspended in the droplet were injected into the on-chip staining region, and the microalgae was stained with the BODIPY followed by incubation in a chamber for complete lipid staining. After that, the droplets were then delivered into the analysis region, where a blue LED was used to simultaneously excite both chlorophyll (red emission) and BODIPY (green emission), and the emitted light was split by a bandpass filter and detected by two PMTs. Chlorophyll fluorescence intensity was used to characterize cell number as a growth indicator, and the BODIPY-stained fluorescence was used to quantify intracellular lipid amount.

Detection methods based on the fluorescence principle have been widely used in microalgal study for cell viability characterization, lipid quantification, species identification and photosynthesis estimation, with a high level of sensitivity and specificity. The chlorophyll fluorescence intensity is proportional to the chlorophyll content, which can be used as an index to evaluate cell activity, growth and photosynthesis effectiveness [95,96,97]. It can also be used to quantify the concentration of living cells in the sample solution, as well as the resistance of microalgae to chemical reagents [98]. As the pigments in different species have different proportions, this method can also be used to classify the microalgae while simultaneously taking into consideration the cell shape and size [78]. The lipid-staining dye can be helpful for estimating the lipid production of the cells, based on which the sorting of high-lipid microalgae on single cell level can be achieved [80,81]. Combining autofluorescence and staining dye fluorescence can simultaneously realize multi-functional detection on a single chip [82,83]. However, the signal from the florescence staining dye can easily be impacted by background fluorescence noise due to autofluorescence and the absorption of the staining dye onto the PDMS. In addition, it is well known that the staining dye will stain all the lipids within the cell, so it is impossible to reflect the types and composition of the lipids.

## 3. Spectroscopic Method for Microalgae Study

As a conventional detection method, Raman spectroscopy can provide chemical identification and quantification of microalgae for label-free and non-destructive measurement [84,99]. Raman spectroscopy is based on the inelastic monochromatic light scattering enabled by a laser in the visible, near infrared or ultraviolet ranges [100]. The Raman effect was first described by the Indian scientist Raman in 1928 [101] and was initially used in biological samples with the introduction of the Fourier-transform Raman instrument in mid-1980s [102,103,104]. Raman spectra can provide specific-chemical analysis of the lipid and pigments according to the molecular bonds of the chemicals. In a typical Raman spectrum diagram, the abscissa indicates the Raman shift and the ordinate indicates the Raman intensity, which can be used to analyze both components and their content [105]. Although the Spontaneous Raman scattering is very weak, improvements in the hardware and techniques have been made to boost the signal of conventional Raman spectroscopy, such as resonant Raman spectroscopy [106], surface-enhanced Raman spectroscopy [107], confocal Raman spectroscopy [108], coherent anti-Stokes Raman spectroscopy [109] and stimulated Raman spectroscopy [110]; these methods effectively enhance the Raman signal and are widely used in microalgae detection [105].

Recently, there have been many investigations on microalgae using off-chip Raman spectroscopy, and the Raman spectrum can reveal the presence and concentration of intracellular chemicals such as beta-carotene chlorophyll, astaxanthin and lipid in different microalgae species [111,112,113]. Brahma et al. proposed a resonance Raman method that can achieve detection and identification of microalgae in water on the basis of differences in chlorophyll and beta-carotene in Raman signals [114]. Collins et al. proposed using confocal Raman microscopy and multivariate analysis for the investigation of pigments in *Chlorophyceae* cells, and this method can acquire the distribution map of beta-carotene, chlorophyll and astaxanthin in three different cellular morphotypes [115]. Daniej et al. studied the intracellular lipid accumulation of *Monoraphidium neglectum* under nitrogen-limited conditions [109]. The results showed that Raman scattering microscopy could also be a reliable tool for directly estimating and quantifying the neutral lipid content for microalgae.

Despite the effectiveness and reliability in microalgae pigments and lipid quantification and characterization using the off-chip spectroscopic method, it still involves manual operation for the manipulation of cells, which is labor-intensive and time-consuming, resulting in a significant decrease in the throughput of the entire test and analysis. Therefore, many researchers have leveraged optofluidic technology to explore the integration of Raman microscope in microfluidic platforms for improvements on the test throughput and the system compactness.

**Figure 2:**
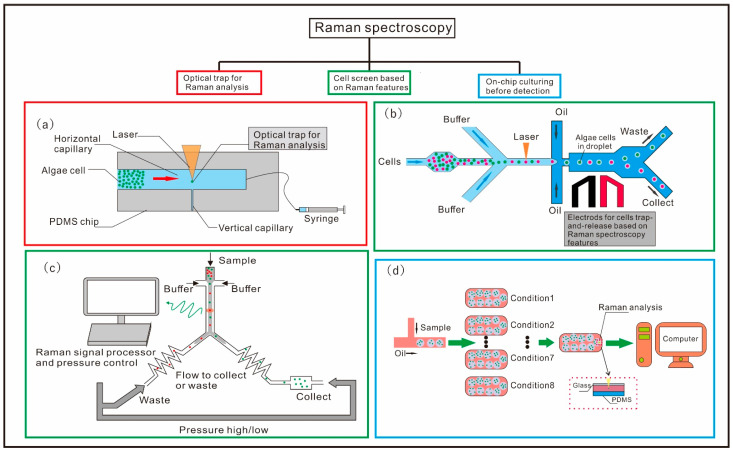
Optofluidic devices for microalgae detection based on Raman scattering. (**a**) Schematic of the microfluidic system for *Trachydiscus minutus* lipid unsaturation detection based on Raman-tweezers sorter [75]. (**b**) Schematic illustration of chip design of the Raman-activated droplet sorting using dielectrophoresis for screening of microalgae single-cells [74]. (**c**) Schematic of the continuous microalgae cell sorting based on resonance Raman spectrum [85]. (**d**) Schematic of the Raman spectroscopy integrated with droplet-based microfluidics [84].

Some researchers have developed droplet-based optofluidic platforms to improve the throughput for cell analysis. Hyun et al. proposed a droplet-based microfluidic for microalgae cell on-chip culture and analysis [84], which can overcome the high Raman background noise from PDMS by inverting the microfluidic chip (Figure 2d), achieving high throughput, time-course tracking and analysis of differential lipid accumulation in microalgae cells under different culture conditions. In this work, the *Chlamydomonas reinhardtii* (*C. reinhardtii*) cells prepared in a medium with eight different nitrogen concentrations were encapsulated in the droplet by a typical T-junction droplet generator and delivered into eight culture chambers for on-chip culturing. The cultured microalgae cells were then analyzed by confocal Raman microscopy. In addition, the lipid content of the *C. reinhardtii* under eight nitrogen concentration conditions was also performed with Nile red staining for estimation of their lipid contents using fluorescence measurement. The comparison between the Raman analysis and the fluorescence detection shows that these two methods have a strong correlation, with R^2^ number up to 0.8614. Wang et al. presented a Raman-activated cell sorting microfluidic system based on the level of astaxanthin content within microalgae cells (Figure 2b), which has a high detection and sorting effectiveness of about 260 cells/min, as well as a high accuracy of 98.3% [74]. Moreover, 92.73% of sorted cells remained alive and were able to proliferate. In this work, the *Haematococcus pluvialis* (*H. pluvialis*) cells in the microchannel were hydrodynamically focused into a single line upon the squeezing of two buffer flows. As the cell passed through the detection region, the content of the astaxanthin in the cell was measured from the Raman spectroscopy, and then the detected cell was encapsulated into the droplet for next-step sorting. Positive dielectrophoresis was used to manipulate the cell in the droplet with efficient trap-and-release, thus forcing cells with different contents of astaxanthin into the pre-designed collection channel or waste channel according to their Raman spectroscopic responses. Besides the dielectrophoresis-based sorting methods, David McIlvenna et al. developed a Raman activated cell sorting system that can realize the cell classification according the carotenoid Raman signal through the control of the pressure in micro-channel [85]. In this work, they integrated a novel microfluidic pressure divider in the chip, aiming to eliminate local pressure fluctuations and provide a stable flow field in the detection region. Carotenoid-containing microorganisms *Synechocystis* sp. were hydrodynamically focused in the detection channel for continuous Raman signal acquisition. If the acquired Raman spectra meet the sorting criteria, the system drives the pump to switch the output pressures to direct the target cell flow to the collection channel. The proposed system can achieve a speed of 0.5 Hz with 96.3% purity of the selected cells. To further improve the throughput, Hiramatsu et al. proposed a flow cytometry integrated with rapid-scan Fourier-transform coherent anti-Stokes Raman scattering spectrometer, which allows stable Raman spectral acquisition with higher efficiency [116]. In this work, the cells were focused into a single line by acoustofluidic force, and the information regarding astaxanthin and chlorophyll was obtained simultaneously to evaluate astaxanthin productivity and photosynthetic dynamics under the condition of nitrogen deficiency with a high throughput of about 2000 events/s.

Raman spectroscopy integrated with microfluidic chip can overcome the limitation of the low throughput of traditional Raman spectroscopy; as a result, numerous devices have been demonstrated to realize the detection and quantification of carotenoid [75,117], astaxanthin [74,76], and lipid content [75,84] in individual cells. The microfluidic platforms combined with Raman microscopy can offer accurate identification and quantification of the composition of the microalgae cell via a label-free, non-destructive, real-time and high-throughput manner. However, the spontaneous Raman signal is relatively weak because of strong Rayleigh scattering. In addition, the autofluorescence from the pigments in the algae cells can overlie the Raman spectrum and lead to misinterpretation during data analysis, limiting the application of this method to some microalgae species with strong autofluorescence intensity [116,118]. As a popular material for the fabrication of microfluidic chips, PDMS can also generate Raman signals that may overwhelm the relatively weak signal from the microalgae cell to be analyzed, so preliminary calibration has to be implemented to eliminate the effect from the PDMS.

## 4. Imaging-Based Flow Cytometry for Algae Analysis

One of the most important challenges is that of realizing rapid and highly efficient identification of a signal microalgae cell group with multiple mixed species and a large sample volume. In the past few decades, many efforts have been devoted to automatic microalgal classification and analysis using flow cytometry, which can be used for species identification, quantitative analysis and the extraction of individual-level parameters [119,120,121]. Generally speaking, a typical flow cytometer can process thousands of cells per minute, which can be much faster than manual observation via optical microscopy [122,123]. However, this kind of conventional cytometer is generally based on the light scattering properties or fluorescence of the microalgae cells, which cannot offer the characterization of the spatial dimensions and distinguish the cells through differences in the cell-surface morphologies [124,125,126]. To overcome this challenge, imaging-based flow cytometry has been introduced for microalgae research, which can offer plenty of information at the single-cell level to differentiate microalgae species on the basis of cell morphology.

To further downsizing the system, microfluidic platforms with optical imaging modules have been introduced for on-chip visualization of microalgae cells with high temporal–spatial resolution [87,127,128]. Benazzi et al. reported a lab-on-chip flow cytometer used for the analysis of the microalgae cells in 2007 [78]. The device was able to simultaneously measure the fluorescence and electrical impedance signals as the algae cells passed through the detection region, and was able to achieve classification and counting of microalgae cells on the basis of the differences in the microalgae cells’ morphological and physiological characteristics. Since then, several fluorescence-based flow cytometers have been developed [129,130].

**Figure 3:**
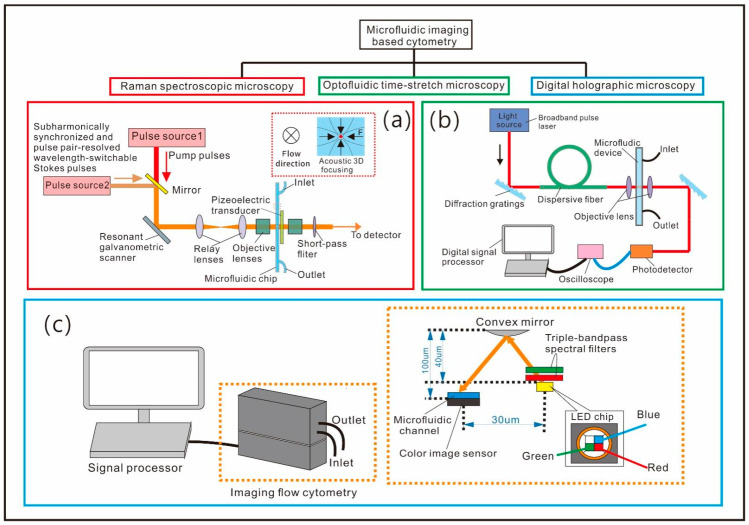
Schematics of the microfluidic imaging-based flow cytometry. (**a**) Schematic of the stimulated Raman scattering imaging flow cytometer [77]. (**b**) Schematic of a flow cytometry based on optofluidic time-stretch microscopy [90]. (**c**) Schematic of imaging-based flow cytometer based on digital holographic microscopy [87].

However, these microfluidic flow cytometry methods strongly rely on the types of microalgae presented and cannot achieve automatic identification of specific species among thousands of microalgae species, as along with the presence of intracellular lipid or pigment. Thus, microfluidic flow cytometry equipped with multi-functional imaging capabilities has emerged for the further investigation of microalgae in a high-throughput, label-free and cost-effective manner. On top of this, image processing algorithms based on machine learning and compressive sensing have been introduced to further improve the throughput and accuracy. Zoltán et al. introduced an imaging-based cytometer using digital holography that can realize real-time imaging of highly dense samples (Figure 3c) [87]. The device can continuously detect the sample cells flowing through a 0.8-mm-thick microfluidic chip with the captured holograms automatically segmented and reconstructed using a deep convolutional network. They then used it for analysis of phytoplankton sampled from the Los Angeles coastline. In the study, 24 different microalgae species were identified in continuously flowing water samples at a high throughput of 100 mL/h. A kind of harmful microalgae *Pseuo-nitzdschia* was identified in six different sampling locations, and the concentration of the microalgae cells was also evaluated. Their results achieved a good agreement with measurement conducted by the California Department of Public Health.

As discussed in Section 2, Raman spectroscopy can provide chemical identification and quantification of the microalgae for label-free and non-destructive detection [84]. The imaging based on Raman scattering can also provide structural information of cells, such as the intracellular mapping of the lipid, protein and pigments [79]. Suzuki et al. proposed high-speed chemical imaging flow cytometry based on multicolor stimulated Raman scattering on a microfluidic platform, which can be used to study the metabolic heterogeneity of microalgae cells (Figure 3a) [77]. The Raman spectroscopic microscopy mainly consisted of a fast pulse pair-resolved wavelength-switchable Stokes laser, a synchronized pump pulse laser and a galvanometric scanner. A three-dimensional acoustic microfluidic chip is used to focus the cells into a single line passing through the detection region. The acquired images containing information of the metabolite’s features were then analyzed on the basis of deep learning methods. The system was able to identify the metabolites of *Euglena gracilis* under nitrogen-sufficient and nitrogen-deficient conditions according to the mapping of three intracellular contents (paramylon, lipids, and chlorophyll) with a high throughput of 140 cells per second. In addition, the results showed an excellent classification accuracy >99%.

CCD or CMOS imaging sensors are necessary for digital holographic imaging cytometry and Raman-activated imaging cytometry; furthermore, the built-in image sensors’ shutter speed and frame rate can be a bottleneck of the system throughput [89]. This challenge has been mitigated by a novel method of optofluidic time-stretch microscopy which can improve the throughput up to 100,000 cells per second. Additionally, the method can also provide near-diffraction-limited spatial resolution and high sensitivity due to its single-pixel detection scheme [131,132]. In 2016, Guo et al. constructed a fluorescence-assisted optofluidic time-stretch microscope consisting of multiple modules including a microfluidic hydrodynamic focusing module, an optical time-stretch microscopy module, and a fluorescence detection module [90]. Optofluidic time-stretch microscopy was used to collect images of the cells in the flow-focusing microchannel, and the fluorescence-assisted analyzer aimed to verify the feasibility of the flow cytometer. The sample cells cultured under nitrogen-deficient conditions were compared with the cells cultured under nitrogen-sufficient conditions, and the results showed that cells stressed by the environment can have a higher lipid content and a lower chlorophyll content. According to the analysis of the acquired images, this multifunctional imaging system can be used to screen *Euglena gracilis* cells under two different culture conditions, reaching a high throughput about 10,000 cells per second with a low false-positive rate of 1.0%. Later, Cheng et al. proposed a label-free high-throughput imaging flow cytometry using optofluidic time-stretch microscopy (Figure 3b) [90], which was mainly composed of a broadband pulse laser, two diffraction gratings and a section of dispersive fiber. The proposed system had a more compact size and fewer optical components. This method basically compared the transparency of the acquired images of the microalgae cultured under different nitrogen conditions using a machine learning classifier trained to differentiate cell types and analyze the same type of microalgae cell that have different lipid content with a relatively high throughput about 1000 cells per second and accuracy 99%.

Leveraging algorithms such as deep learning and compressive sensing, microfluidic imaging-based flow cytometry could serve the powerful function of automatically identifying and counting microalgae, with a high throughput of up to thousands of cells per second and with high accuracy. Especially enabled by novel multi-functional imaging techniques, the microfluidic flow cytometer can offer information regarding both cell morphology and the intracellular lipid and pigment. Compared with microfluidic devices based on fluorescence detection and Raman spectroscopy, the imaging-based flow cytometer has flexibility and feasibility for realizing machine automation without human intervention. On the other hand, automatically identifying and classifying the microalgae cell requires a database for machine training, and thus highly experience-based preparatory image requisition of samples has to be implemented before on-site usage.

## 5. Conclusions

Considering that microalgae is widespread in rivers, lakes and oceans and is closely related to human health, environmental protection and economics, it is essential to develop devices that are rapid, cost-effective and portable in order to monitor the dynamics of the microalgae. As optical detection techniques have the merits of high-precision, fast, and non-contact measurement, many researchers have explored the integration of optical instruments and various microfluidic platforms for microalgal detection. So far, the developed optofluidic devices and systems can successfully achieve microalgal characterization and quantification such as cell activity, pigment and lipid content, as well as their metabolic heterogeneity evaluation. The optofluidic platforms based on fluorescence detection have been most widely favored, and can realize cell activity detection, counting, microalgae species identification, growth and photosynthetic efficiency estimation as well as lipid quantification. The current challenge with this method lies in the fact that some microalgae species are resistant to the staining dye. Furthermore, the environmental light could introduce perturbation into the fluorescence measurement, making the method difficult for in situ detection. The optofluidic systems based on Raman spectroscopy are mainly used to detect and quantify the intracellular substances of microalgae cells. It has the capacity to provide finger-print information of the compositions according to the special molecular bond, which does not require any labeling procedure, and can be applied in any microalgae species, including fluorescence-dye-resistant microalgae species. However, the relatively weak spontaneous Raman signal needs more complicated optical instruments to ensure robust acquisition of data to avoid the interference from the PDMS and sample solution. In addition, the data acquisition process of Raman spectra could be a bottleneck of the analysis throughput of the entire system. Optofluidic imaging-based flow cytometers can achieve a higher throughput, and can provide precise analysis and statistics of microalgae species on the basis of their morphological properties. In addition, this method can be extended by hybridization with new optical imaging methodologies, such as photoacoustic microscopy [133,134], holographic microscopy [135,136], time-stretch microscopy [88,90], etc., to realize multi-functional parameter extraction.

Photoacoustic imaging is a technology based on laser-generated ultrasound. When incident light illuminates the light-absorbing material, the material absorbs the light and partly converts the energy into acoustic waves. Photoacoustic imaging exhibits excellent advantages, such as rich optical contrast, deep penetration depth, and specific ability of three dimensional (3D) imaging, which guarantees the wide use of visualization and detection of biological tissues in in vitro and in vivo clinical studies [137,138]. Song et al. developed a microfluidic photoacoustic microscopy technique that realized both 2-dimensional (2D) and 3-dimensional visualization of the droplets with high throughput [133]; the lateral resolution of this technique can be enhanced to reach an optical diffraction limit (~5 μm). Subsequently, the opto-acoustic-fluidic-based technology was used for the detection and identification of red blood cells and circulating primitive cells [139]. However, to the best of our knowledge, only limited studies have applied photoacoustic in single plant cells [140,141]; considering the rich content and types of pigments inside microalgae cells, we believe that microfluidic-based photoacoustic microscopy must have a variety of applications in microalgae identification, cell viability estimation and qualitative analysis of pigments or lipid.

Digital holography (DHM) is an interferometric imaging technique that can record both amplitude and phase information of the wave front diffraction of the object [142,143]. There are two main types of configurations in digital holography: off-axis DHM and on- axis DHM. Generally speaking, on-axis DHM is often used for 3D position tracking of an object, and off-axis DHM is a powerful tool for 3D visualization of samples with a sub-micron resolution along the axial direction. Luo et al. developed a real-time quantitative phase microscopy that can provide 3D information and chemical parameters of the flowing droplets with high optical resolution (axis: 77 nm, lateral: 0.9 µm) [67]. Current microfluidic-based DHM platforms and traditional imaging methods in microalgae research focus on 2D morphology analysis but lack 3D imaging and intracellular chemical analysis. We hold the opinion that off-axis DHM may be a powerful tool for microalgae 3D morphology analysis and intracellular substance visualization. Additionally, on-axis DHM-reflected 3D position information may also have significant meaning in environment-related research, because some swimming microalgae usually change their position in accordance with the surrounding environment, which can be used as a biosensor for water quality monitoring and toxicity warning [144,145].

Time-stretch imaging is a revolutionary imaging method that can temporally stretch broadband pulses by using the dispersive properties of light in both spatial and temporal domains, and it can image cells at a high frame and shutter time, improving the throughput to up to 100,000 cells per second [146,147,148]. Optofluidic time-stretch microscopy-based imaging cytometry applied in microalgae species significantly improves detection throughput with high accuracy [95,96,97]. If this kind of technology can realize the sorting function, it will be beneficial for microalgae strain screening and cultivation, thus improving the productivity of the lipid, pigments and polysaccharides, and promoting the commercial application of the microalgae [149].

In a word, the development of microfluidic platforms for microalgae detection should lead to a successful commercialization of microalgae, which can not only provide extracellular and intracellular information, but also sort target cells accurately and immediately. Optofluidic detection methods provide multiple methods that can simultaneously realize the microalgae cell detection and manipulation. In this paper, we introduced three optofluidic technologies based on fluorescence, Raman spectroscopy and imaging-based flow cytometry applied in microalgae detection and characterization. Then, we analyzed the advantages and disadvantages of these methods. Finally, the outlook of the optofluidic detection platform was investigated and discussed.

## Figures and Tables

**Table 1:** Applications of the microfluidics for microalgal detection and quantification.

Applied Species	To Be Detected/ Function	Throughput	Detection Method	Light Source	Sensing Principle	Ref.
Six species ^(a)^	Cell viability and counting	NM ^(b)^	Autofluorescence	A 488 nm laser	Chlorophyll fluorescence	[71]
Four species ^(c)^	Algae identification and counting	~100 cells/min	Autofluorescence	A 488 nm and a 633 nm laser	Chlorophyll, phycoerythrin and cells’ shape	[73]
*Dunaliella salina* and *Tetraselmis Chui*	Cell viability	NM	Autofluorescence	A 488 nm laser	Chlorophyll fluorescence	[72]
Three species ^(d)^	Algae identification	~100 cells/min	Autofluorescence and electrical impedance	A 532 nm and a 633 nm laser	Fluorescence properties ^(e)^ and electrical impedance	[78]
Three species ^(f)^	Growth dynamics and cells screen	NM	Autofluorescence	A 488 nm laser	Chlorophyll Fluorescence	[79]
Three species ^(g)^	Lipid content and cell viablity	NM	Fluorescence dye staining	No laser (Fluorescence microscope)	BODIPY fluorescence dye and SYTOX fluorescence probe	[80]
*Neochloris oleabundans*	Lipid content	NM	Fluorescence dye staining	No laser (Epifluorescent microscope)	BODIPY fluorescence dye	[81]
*Chlamydomonas reinhardtii* and *Botryococcus braunii*	Growth and lipid content	~300 cells/min	Autofluorescence and fluorescence dye staining	A blue LED	Chlorophyll fluorescence and BODIPY fluorescence dye	[82]
*Phaeodactylum tricornutum*	Photosynthetic efficiency and lipid accumulation	~6000 cells/min	Autofluorescence and fluorescence dye staining	A 785 nm laser and a 470 nm LED	Chlorophyll fluorescence and Nile Red fluorescence	[83]
*Saccharomyces cerevisiae*	Carotenoid content	~240 cells/min	Raman spectrum	A 532 nm laser	Molecular bond	[76]
*Haematococcus pluvialis*	Astaxanthin content	~260 cells/min	Raman spectrum	A 532 nm laser	Molecular bond	[74]
*Chlamydomonas reinhardtii* and *Botryococcus braunii*	Lipid content	NM	Raman spectrum	A 532 nm laser	Molecular bond	[84]
*Synechocystis* sp.	Carotenoid content	~120 cells/min	Raman spectrum	A 532 nm laser	Molecular bond	[85]
*Trachydiscus minutus*	Lipid unsaturation	~6 cells/min	Raman spectrum	A 785 nm laser	Molecular bond	[75]
*Haematococcus lacustris*	Astaxanthin content and photosynthetic dynamics	~2000 cells/s	Raman spectrum	A pair of femtosecond laser	Molecular bond	[86]
*Pseudo-nitzschia*	Cell identification and counting	~5000 cell/min	Digital holographic microscopy	Three LEDs	Morphology difference	[87]
*Euglena gracilis*	Metabolic Heterogeneity ^(h)^	~8400 cells/min	Raman-activated imaging microscopy	A wavelength switchable laser and a synchronized pump pulse laser	Molecular bond	[77]
*Scenedesmus* and *Chlamydomonas*	Cells classification and counting	NM	Optofluidic time-stretch imaging microscopy	A broadband pulsed laser	Morphology difference	[88]
*Euglena gracilis*	Cell identification and counting	~10,000 cells/s	Optofluidic time-stretch imaging microscopy	A broadband pulse laser	Morphology difference and fluorescence signal	[89]
*Euglena gracilis*	Screen cells and number statistics	~10,000 cells/s	Optofluidic time-stretch imaging microscopy	Sapphire femtosecond pulse laser and a 488 nm pulse laser	Morphology difference	[90]

^(a)^ Karenia mikimotoi Hansen, *Chlorella vulgaris*, *Nitzschia closterium*, *Platymonas subcordi formis*, *Pyramidomonas delicatula* and *Dunaliella salina*. ^(^^b)^ NM means not mentioned. ^(c)^ Karenia b., *Synechococcus* sp., Pseudo-Nitzchia and Alexandrium. ^(d)^ *Isochrysis Galbana*, Rhodosorus. m and *Synechococcus* sp. ^(e)^ Pigments fluorescence of chlorophylls, phycoerythrin and allophycocyanine. ^(^^f)^ *Synechocystis* PCC 6803, Synechococcus PCC 7002 and *Chlamydomonas reinhardtii*. ^(g)^ *Chlorella vulgaris*, *Chlamydomonas* sp. and *Botryococcus braunii*. ^(h)^ Microalgae culturing in different nitrogen condition have different content of chlorophyll, paramylon and lipids.
